# Editorial: The use of emerging technologies in occupational health and safety, volume II

**DOI:** 10.3389/fpubh.2023.1200044

**Published:** 2023-06-13

**Authors:** Luis Möckel, Hamzeh Mohammadi, Somayeh Farhang Dehghan

**Affiliations:** ^1^IU Internationale Hochschule GmbH, University of Applied Sciences, Düsseldorf, Germany; ^2^Faculty of Health and Medical Engineering, Tehran Medical Sciences, Islamic Azad University, Tehran, Iran; ^3^Environmental and Occupational Hazards Control Research Center, School of Public Health and Safety, Shahid Beheshti University of Medical Sciences, Tehran, Iran

**Keywords:** occupational health, chatbot, ergonomic, text-based, burnout

Emerging technologies and their application in the cycle of daily life and business will bring new opportunities in front of us and double our mental and physical abilities. Humanity today, relying on such developments, will be able to go beyond the current scale and boundaries and present new achievements. Therefore, increasing the mental and physical power of man and improving his living standards can be considered as the most important application of new technologies, which itself will lead to the emergence of newer technologies and will exponentially pave the way for more progress. All branches of different sciences and their progress depend on the growth and development of modern technology, and the current world cannot be imagined without them.

Occupational Health and Safety can be briefly defined as the science and art of providing health in workplaces, and an Occupational Health Professional is someone who is concerned primarily with the control of environmental stresses or occupational health hazards that arise because of or during work. The use of emerging technologies in Occupational Health and Safety can affect various aspects of this science and can make its high goals, which are the anticipation, recognition, evaluation, and control of these environmental factors or stresses arising in or from the workplace, more achievable.

The Editorial presented the contributing articles of the Research Topic of “*The use of emerging technologies in occupational health and safety, volume II*”, related to Section Occupational Health and Safety of Frontiers in Public Health, which were published from September 2022 to March 2023. The purpose of the Editorial was to frame the objectives of the research within the topic, as well as place its findings in a broader context. The Research Topic has published six articles from countries such as China, Germany, Spain, the UK, and Iran about the following topics:

- Predicting occupational injury causal factors using text-based analytics,
- Developing a chabot for construction safety training,
- Cybergonomics,
- Aerosol formation when using cobas e analyzers,
- The use of Digital transformation to mitigate Burnout Syndrome, and
- Evaluation of the ergonomic risk of clinical physiotherapy practice.

Khairuddin et al. reviewed text mining and natural language processing (NLP) for occupational injury reports. The study used advanced deep learning and automated text analytics to extract occupational injury narratives. The authors evaluated the usefulness of these techniques for predicting occupational injuries. They found a gap in the literature and proposed their own framework for the study. NLP models with machine and deep learning techniques show promise in studying occupational injury narratives. They can classify accidents, identify the causes of injuries and predict injury rates. This study identified constraints in injury report methodologies, categorized under three headings, including inconsistent terminology and abbreviations leading to misclassification of text. Limited sample sizes and distinct populations reduce model generalizability due to concerns about data adequacy and authenticity. The imbalanced data distribution affects analysis complexity. It is crucial to tackle these hurdles for better text analysis deployment. Workplace injuries can harm employee health and create financial burdens for both employees and employers due to direct and indirect costs. Disruptive changes in workers' psychological and behavioral states can harm productivity.

We must analyze occupational injury to reduce mishaps in work settings, using data from injury reports. Previous studies show that using big data technology and machine learning outperforms traditional statistical models in predicting future events. Analyzing and forecasting occupational accidents and injuries is vital for identifying their impact on society. These patterns can improve safety protocols and intervention methods for industry stakeholders. Corporations track and document work-related injuries and illnesses, which can be analyzed and converted into numerical data. Predictive modeling tools can recognize scoring patterns. Although it provides various facts about work accidents, this info is essential.

Zhu et al. created four chatbots on a Telegram for safety training in construction used in an experiment as an intervention. This study examined AI-based online eye-tracking technology for evaluating construction hazard awareness. The study analyzed the effect of using a Telegram chabot on improving hazard awareness in construction workers. They analyzed safety awareness data from a full factorial experiment to determine the factors affecting the effectiveness of Telegram chabot safety training. This study enhances the knowledge of using technology and innovative methods to improve construction site safety. They found that using a chabot for safety training improved hazard awareness among inexperienced workers. The chabot intervention showed effectiveness for 5 days, even during the Ebbinghaus' forgetting curve. A cheap Chabot training could increase site workers' awareness of hazards. Design the training program to fit participants' construction site experience and employment status considering the financial and logistical challenges of classroom-based training and the shift to remote learning due to COVID-19, shown by the increase in virtual education platforms such as Coursera, EdX, and online degrees from universities. This study suggests that adding a Telegram chabot can supplement hazard awareness training.

Safety training boosts risk perception and hazard awareness in construction, and the effectiveness of safety measures is essential for occupational health. Due to lockdown in pandemics such as COVID-19, face-to-face safety training is being overshadowed, leading the authors to consider other methods. A chabot is a messaging software that enables human-computer interaction through an algorithm, for information retrieval, enquiries, and sales management. Chatbots have been used in language education, but more research is needed to determine their effectiveness in increasing hazard awareness after training programs.

The next article discusses Pouyakian's theory on the history and development of ergonomics and its subfields sequential. Pouyakian's Cybergonomics is a new concept for applying ergonomics to industry 4.0. He defines Cybergonomics thoroughly ergonomics, or human factors, emerged due to the industrial revolution. Ergonomics has advanced alongside the development of branches focused on human physical, cognitive, and organizational function. The fourth industrial revolution has brought cyber-technologies into everyday life, leading to the emergence of cyber-elements in different domains. Applied disciplines have adapted to cyberspace, incorporating the cyber-prefix into their terminology. Cybernetics studies self-regulating systems, including biology and mechanics. ‘Cyber' denotes internet-related ideas and modern systems. “Cyber” encompasses various terms such as computerized, networked, and high-tech, among others. Based on this, the author suggests a new term, Cybergonomics. A new word “cyberergonomics” has been formed by blending “cyber” and “ergonomics” with “er” as the common element. This refers to the ergonomics of human-cyber interface in digital lifestyles and work. Cybergonomics study in progress, lots of online sources for literature. The research has been widely shared in computer science and engineering publications, including “Robotics and Computer Integrated Manufacturing,” “Procedia Computer Science,” “Procedia Engineering,” and “Technological Forecasting & Social Change.” The authors of these studies focus on cybernetic components and attract computer science journals due to vested interest. Pouyakian proposed a research plan for the emergence of Cybergonomics. Also, he linked Cybergonomics with Industry 5.0 goals. Cybergonomics can help achieve Industry 5.0 goals by protecting people from the negative effects of new tech and improving high-tech workforce interaction through necessary regulations and adaptations.

The aerosol generation when manipulating model samples in a laboratory was evaluated by Burghardt et al., specifically the use of cobas e analyzers alone or with an integrated workflow. The findings were used to examine the risk of lab operators from SARS-CoV-2, using HBsAg as a proxy for virus particles. Air sampling at multiple locations using the Elecsys^®^ HBsAg II II quant immunoassay-quantified HBsAg aerosol formation. Findings showed that the cobas e analyzers had an average HBsAg uptake of 1.9 viral particles per hour, compared to 0.87 viral particles in the overall laboratory workflow. Max inhalation rate < 16 viral 24-h shift. The study found that using cobas e analyzers and an end-to-end workflow produces few marker-bearing aerosols. Lab operators may be at a risk of contracting SARS-CoV-2. The COVID-19 is caused by SARS-CoV-2 and testing for it helps reduce transmission. Lab personnel are essential in administering SARS-CoV-2 tests during the pandemic. Lab operators face occupational risk from potential exposure to infected specimens of SARS-CoV-2. Recombinant HBsAg is a suitable surrogate since research has shown that it can form virus-like particles easily dispersed through the air. It is suitable for comparative study with other airborne infectious agents. The HBsAg and SARS-CoV-2 viral particle sizes are comparable.

Sanchez-Segura et al. in a policy brief article analyzed how Burnout Syndrome and digital technologies have increasingly intersected in modern occupations. They evaluated digital tech's ability to ease Burnout Syndrome. They indicated that creative strategy is essential for Burnout Syndrome treatment, so traditional methods may not produce unique results. This study explores an innovation contest aimed at using technology to address Burnout Syndrome. Twelve innovative projects were conceptualized with practical financial plans, including analysis, design, and management. The goal was to achieve efficiency by implementing the proposed idea. This text highlights IRSST (Instituto Regional de Seguridad y Salud en el Trabajo)'s innovative initiatives in occupational health and safety (OHS) in Madrid, Spain. This highlights the expected impact of these projects on improving the OHS. 8 out of 12 proposed mobile apps, 4 suggested website-based approach, and another 4 suggested an independent approach.

The success of the proposed solutions and their adoption by users depend on an efficient and user-friendly tool that respects their daily routines. Lessons learned: Simple and user-friendly mechanical layouts, particularly for multi-user and cross-platform web applications, are most effective. Joining established strategies for treating burnout, offers valuable insights for future planning. Making a unique presentation to appeal to customers and professionals.

Burnout disorder affects 10% of professionals due to misplaced efficiency and sick leave expenses. Burnout signs are easy to detect, but their impact is hard to measure, posing risks for businesses such as employee unhappiness, less effective, and lower quality of life. Many non-routine methods exist for personal self-management and well-being; however, but few are used at the organizational level to prevent burnout. This investigation resolved the shortage of official devices by making them more accessible.

Fan et al. assessed ergonomic risk in clinical physiotherapy using motion analysis and Rapid Entire Body Assessment (REBA) scale. Structured light sensors and subjective measures of Perceived Physical Exertion (RPE) were used to evaluate physical exertion. A tailored ergonomic assessment program was tailored to physiotherapists' needs, with high accuracy. This involved including relevant behavior aspects for physiotherapists. They created an automated tool for ergonomic evaluations designed for physiotherapists. Previous studies have focused on work-related musculoskeletal diseases (WMSD) experienced by physiotherapists. The ergonomic evaluation detects hidden musculoskeletal disorders, and interventions based on ergonomic principles prevent work-related injuries. This method is commonly used in surgery to evaluate ergonomic factors during complex procedures. Studying ergonomic risk factors in clinical physiotherapy can help prevent injuries to physiotherapists, boost job satisfaction, and improve patient care.

The articles published in the Research Topic clearly indicate how we benefit from emerging technologies in the occupational health and safety. Technologies are used to predict occupational injury outcomes with deep machine learning and text-minig, to automatically assess and identify ergonomic risk factors in physiotherapists, and to analyze the exposure risk of laboratory workers to infective agents. Additionally, Chabot technologies are used to train workers on safety risks in the construction industry. But it must be mentioned here that the technologies itself can become occupational risk factors. Therefore, cyberergonomics and the mitigation of technology -related burnout can be considered as highly relevant current and future topics. The articles published in Volume II of Research Topic “*The use of emerging technologies in Occupational Health and Safety*” provide a valuable overview on how technologies can help reduce occupational hazards and increase safety and health in various occupations.

## Author contributions

All authors listed have made a substantial, direct, and intellectual contribution to the work and approved it for publication.

